# Quality of life in people with diabetes: a systematic review of studies in Iran

**DOI:** 10.1186/2251-6581-12-54

**Published:** 2013-12-19

**Authors:** Aliasghar A Kiadaliri, Baharak Najafi, Maryam Mirmalek-Sani

**Affiliations:** 1 Division of Health Economics, Department of Clinical Sciences, Malmö University Hospital, Lund University, Malmö, Sweden; 2 Department of Health Management and Economics, School of Public Health, Tehran University of Medical Sciences, Tehran, Iran; 3 Health Economics & Management, Institute of Economic Research, Lun University, Lund, Sweden; 4 Mehregan Parto Research Institute, Tehran, Iran; 5 International Cooperation Affairs, Deputy of Social Welfare, Ministry of Cooperatives, Labour & Social Welfare, Tehran, Iran; 6 Lund University, Department of Clinical Sciences, Malmö, Sweden; 7 Skane University Hospital, 20502 Malmö, Sweden

**Keywords:** HRQoL, Diabetes, Systematic review, Iran

## Abstract

Evaluation of health-related quality of life (HRQoL) among people with diabetes has been growing in Iran over the last decade. The main aim of the current study was to systematically review the characteristics of these studies and examine quality of their findings. Persian (SID, Magiran) and English (Pubmed, Medline, Web of Science, CINAHL, Scopus, PsycINFO and ERIC) databases were systematically searched using the search terms: “diabetes” AND “quality of life” AND “Iran”. The Preferred Reporting Items for Systematic Reviews and Meta-Analyses (PRISMA) guidelines were followed. A total of 46 studies passed the inclusion criteria and were included in the review. The included studies were conducted in 20 out of 30 provinces of the country. Most studies investigated HRQoL among people with type 2 diabetes. The Short Form Health Survey (SF-36) and WHO quality of life instruments (WHOQOL) were the main instruments used in these studies. Studies showed that people with diabetes had lower HRQoL than people without diabetes. Better socioeconomic status and better control of cardiovascular risk factors were associated with better HRQoL among the patients with diabetes. In general, the predictors of HRQoL among Iranian patients were similar to their international counterparts implying that diabetes patients share many common features. The reviewed studies suffer from major methodological and reporting flaws which limit validity and generalizability of their findings.

**Electronic supplementary material:**

The online version of this article (doi:10.1186/2251-6581-12-54) contains supplementary material, which is available to authorized users.

## Introduction

Due to insufficiency of traditional end points (which are mainly focused on the biologic and physiologic outcomes) in capturing the effects of interventions on patients’ health-related quality of life (HRQoL), a growing interest has emerged during the past decades for assessing determinant factors of patients’ HRQoL, especially in chronic diseases [1]. Diabetes mellitus is one of these chronic diseases that involve people of all ages and races. It is considered as one of the most common chronic diseases in approximately all countries, and its prevalence continues to increase mainly due to the changes in lifestyles resulting in physical inactivity, and increased obesity [2]. It was estimated that diabetes affected 285 million adults (20–79 years) worldwide in 2010, and this figure will increase to 439 million adults by 2030 [2].

Diabetes is associated with higher risk of some macro and microvascular complications. As result, these complications cause mortality rate among diabetic patients to be about twice as much as that of non-diabetic individuals of a similar age [3, 4]. Moreover, patients with these complications have lower HRQoL than diabetes patients without the complications [5, 6].

In Iran, prevalence of diabetes increased from 7.7% in 2005 to 8.7% in 2007 [7, 8]. In addition, it was estimated that annual direct medical cost of diabetes is roughly US$ 113 million and direct medical cost in patients with diabetes is about 3 times higher than general population in the country [9]. High prevalence of diabetes and its related complications have attracted the research and policy concern in the country over last few years. In response to this policy concern, a considerable body of literature has been emerged to evaluate HRQoL and its determinants in diabetic patients. These studies aimed to improve HRQoL in people with diabetes by providing evidence for informed decision-making. However, differences in the research questions, tools and population among these individual studies make it difficult to reach an obvious answer applicable for policy making purposes. In response to this, conducting a systematic review of individual studies to make the available evidences more accessible for policy-making is common in medical researches.

In this course, the current systematic review was conducted to describe the latest available information about HRQoL in people with diabetes in Iran. Specifically, this review aimed to investigate how HRQoL was measured in Iranian diabetic population, what were the main methodological flaws of these studies, and which factors were mainly associated with HRQoL in people with diabetes.

## Method and materials

### Literature search

A systematic literature search was independently conducted in March 2012 to review the studies which evaluated HRQoL among people with diabetes in Iran. The results of this literature search were independently verified and updated in June 2012. Studies published up to May 2012 were included in the review. National (SID, Magiran) and international databases (Pubmed, Medline, Web of Science, CINAHL, Scopus, PsycINFO and ERIC) were searched through following search terms: [“diabetes” AND “quality of life” AND “Iran”]. Moreover, we searched Google for extra Persian publications. We followed the Preferred Reporting Items for Systematic Reviews and Meta-Analyses (PRISMA) guidelines [10].

### Selection of studies

Five exclusion criteria were applied: (1) the study did not investigate HRQoL in people with diabetes, (2) the study did not provide any data about HRQoL among study population (e.g., the study was related to instrument development in patients with diabetes); (3) the study was a review article; (4) the study was not a journal article (e.g., conference abstracts and dissertations); (5) the study was not applied to the Iranian population.

**Figure 1:**
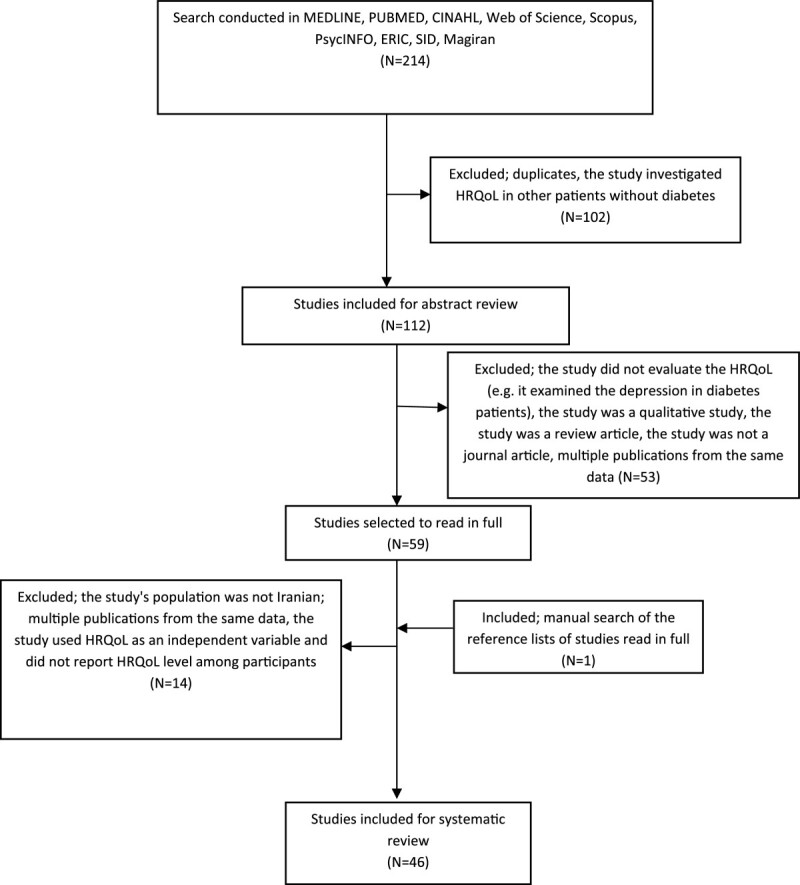
**Flow diagram of the literature search.**

The initial search resulted in 214 documents. After excluding duplicates and non-relevant studies, 59 articles were selected for full text examination. The reference lists of these 59 documents were manually searched. In total, 46 studies were included for the review (Figure 1). In cases where multiple publications were produced from a single study, the paper with most comprehensive data was included.

Data extracted from the selected studies are included, among others: year of publication, the location (or province) and the year the studies were conducted, study design, sample size, type of diabetes, age range of the sample, duration of diabetes, HRQoL measurement tool used, main predictors of HRQoL, and statistical methods used for analysis.

## Results

### General characteristics

**Figure 2:**
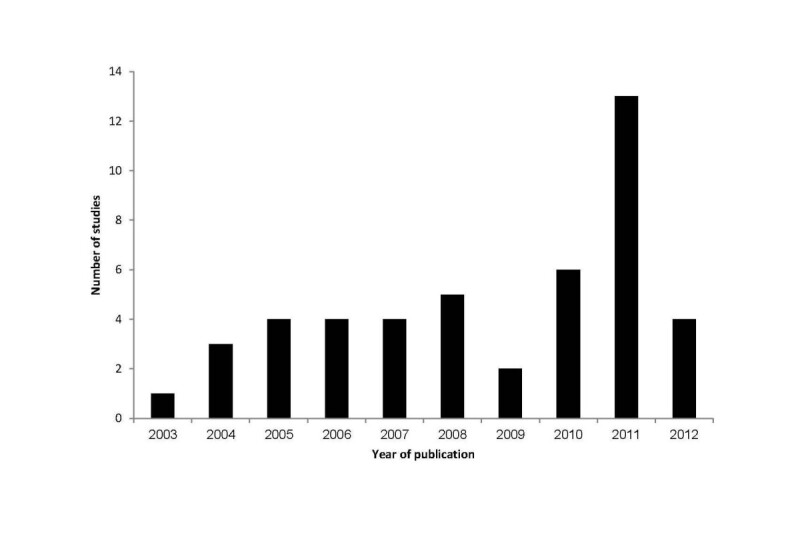
**Number of studies by year of publication.**

The characteristics of 46 eligible studies [11–56] for this review are shown in Table 1. First study was published in year 2003 and since then there were few publications per year on the topic, with a peak in the publications in 2011 (Figure 2). HRQoL in people with diabetes was investigated in 20 out of 30 provinces of the country. 17 out of 46 studies were conducted in two provinces (Tehran & Hormozgan). Sample sizes across the studies ranged from 27 to 387 participants. Majority of the studies (76%) were published in a national Persian language journal. Cross-sectional design was the dominant design in the studies (N=32). In terms of type of diabetes, five studies included only people with type 1 diabetes and 23 studies covered only type 2 diabetic patients. In the remaining studies, there was a combination of type1, type2, and non-diabetes people in the sample. The age of participants ranged from 10 to 75 years old. In most studies, women constituted the majority of the study participants.

**Table 1:** **Characteristics of the studies included in the review**

First author	Data year	Language	Province	Main interest variable	Study design	Sample characteristics					HRQoL assessment		Statistical analysis
						Type of diabetes (%)	Sample size	Age range	Females (%)	Diabetes duration (mean/median)	Instrument	Specific or generic?	Adjusted (A) or unadjusted (U)?
Aghamolaei T, 2003 [11]	-	English	Hormozgan	Predictors of HRQoL	Cross sectional	Type 2 (100)	80	32-72	58.8	5.8	WHOQOL-BREF	Generic	U
Aghamolaei T, 2005 [12]	-	Persian	Hormozgan	Health education	Quasi-experimental	Type 2 (100)	71	-	59.2	6.0	WHOQOL-BREF	Generic	U
Ahari SS, 2008 [13]	2004	Persian	Ardebil	Type 2 diabetes	Cross-sectional	Type 2 (100)	110	-	66.4	8	SF-36	Generic	U
Ahmadi A, 2011 [14]	2008	Persian	Chaharmahal Bakhtiari	Predictors of HRQoL	Cross-sectional	Type 2 (100)	254	30-65	59	7.4	Developed by research team	Specific	A
Alavi A, 2010 [15]	2008	Persian	Chaharmahal Bakhtiari	Type 1 diabetes	Historical cohort	Type 1 (14.5), non-diabetes (85.5)	152	8-18	52.4 in diabetes group	NA	PedSQL	Generic	U
Alavi NM, 2004 [16]	2003	Persian	Tehran	Predictors of HRQoL	Cross-sectional	Type 1 (13),Type 2 (87)	104	>18	65	9.7	Developed by research team, Well Being Index	Specific, generic	U
Bagheri H, 2005 [17]	NA	Persian	Semnan	Micro-& macrovascular complications	Cross-sectional	Type 2 (100)	150	35-65	NA	NA	Audit of Diabetes Dependent Quality of Life	Specific	U
Baghianimoghadam MH, 2007 [18]	NA	Persian	Yazd	Health education	Quasi-experimental	Type 2 (100)	120	25-75	59	9.87	SF-20	Generic	U
Bazzazian S, 2010 [19]	NA	Persian	Tehran	Coping strategies	Cross-sectional	Type 1 (100)	300	18-30	57.3	NA	D-39	Specific	A
Borzou SR, 2010 [20]	NA	Persian	Hamedan	HRQoL level	Cross-sectional	Type 2 (100)	165	NA	67.3	NA	SF-36	Generic	U
Delvarianzadeh M, 2006 [21]	NA	Persian	Semnan	Diet consultation	RCT	Type 2 (100)	144	35-65	67.8	NA	SF-36	Generic	U
Farahani TS, 2010 [22]	2007	Persian	Tehran	Age and sex	Cross-sectional	Type 1 (100)	70	11-18	52.1	2.23	Diabetes Quality of Life for Youth	Specific	U
Ghanbari A, 2004 [23]	2000	Persian	East Azerbaijan	Predictors of HRQoL	Quasi-experimental	Type 2 (100)	137	NA	86.1	NA	NA	Generic, specific	A
Ghanbari A, 2004 [24]	NA	Persian	Gilan	Type 2 diabetes	Cross-sectional	Type 2 (51.1), non-diabetes (48.9)	176	>40	78.8% (diabetic group), 55.8% (non-diabetic group)	NA	SF-36, SWED-QUAL	Generic	A
Ghanbari A, 2005 [25]	2000	English	East Azerbaijan	Predictors of HRQoL	Cross-sectional	Type 2 (100)	117	>35	85.5	NA	SWED-QUAL, diabetes-specific quality-of-life scale	Generic, specific	A
Ghavami H, 2005 [26]	2003-2004	Persian	West Azerbaijan	Continious care	Quasi-experimental	Type 2 (100)	74	40-65	NA	NA	Developed by research team in Iran (Alavi NM)	Specific	U
Haririan H, 2009 [27]	2007	Persian	East Azerbaijan	Aspects of HRQoL	Cross-sectional	Type 2 (100)	150	20->60	61.33	NA	SF36+Swed- QUAL, a diabetes-specific questionnaire	Generic, specific	A
Hashemi Hefzabad F, 2011 [28]	NA	English	Isfahan	Diabetes impact on HRQoL	Cross-sectional	Diabetes (50), non-diabetes (50)	204	20-60	52% in both groups.	NA	Hanestad & Albrektsen’s Attitude to Quality of Life	Generic	U
Heidari M, 2007 [29]	2004-2005	Persian	Zanjan	Empowerment model	Quasi-experimental	Type 1 (100)	47	11-20	-	-	-	-	U
Jafari P, 2011 [30]	NA	English	Fars	Impact of type 1 diabetes on HRQoL	Cross-sectional	Type 1 (32), healthy (68)	294	8-18	56.4% in diabetes and 53% in healthy group.	NA	PedsQL™ 4.0 Generic Core Scales, PedsQL™ 3.0 Diabetes Module	Generic, specific	A
Jahanlou AS, 2007 [31]	2006	Persian	Hormozgan	Smoking	Cross-sectional	Type 2 (100)	125	NA	NA	NA	WHOQOL-BREF 26	Generic	U
Jahanlou AS, 2008 [32]	2007	Persian	Hormozgan	Glycemic control	Cross-sectional	Type 2 (100)	110	27-72	66.9	6.33	WHOQOL-BREF 26	Generic	U
Jahanlou AS, 2011 [33]	2007	English	Hormozgan	Education	Cross-sectional	Type 2 (100)	256	27-72	67.5	6.33	WHOQOL-BREF 26	Generic	U
Jahanlou AS, 2011 [34]	2006	English	Hormozgan	HbA1c	Cross-sectional	Type 1 (11.8), Type 2 (88.2)	76	NA	60.5	NA	WHOQOL-BREF 26, Iranian Diabetics’ Quality of Life	Generic, specific	U
Jahanlou AS, 2011 [35]	2007	English	Hormozgan	-	Cross-sectional	Type 2 (100)	387	27-72	51.9	5.83	WHOQOL-BREF 26, Iranian Diabetics’ Quality of Life	Generic, specific	U
Kakhaki AD, 2006 [36]	2004	Persian	Tehran	Predictors of HRQoL	Cross-sectional	Type 1 (15.3), Type 2 (84.7)	131	18-65	60.3	6.10	SF-36	Generic	U
Kasbakhi MS, 2008 [37]	2008	Persian	Mazandaran	Type 2 diabetes	Case–control	Type 2 (48.3), non-diabetes (51.7)	145	NA	90% in diabetic group and 81.3% in non-diabetic group.	NA	SF-36, SWED-QUAL	Generic	U
Kermansaravi F, 2012 [38]	2011	Persian	Sistan & Baluchestan	Predictors of HRQoL	Cross sectional	Type 1 (100)	100	10-18	47	3.5	Diabetes quality of life youth	Specific	U
Khaledi S, 2011 [39]	2009	Persian	Kordestan	Predictors of HRQoL	Cross-sectional	Type 2 (100)	198	>18	83.8	NA	SF-36	Generic	U
Khamseh MA, 2011 [40]	2009-2010	Persian	NA	Aspects of HRQoL	Cross-sectional	Type1 (100)	150	12-30	49.3	8.97	Developed by research team	Specific	A
Nejatisafa, 2008 [41]	2005	Persian	Tehran	HbA1c	Cross sectional	Type 1 (4), Type 2 (96)	100	18-65	68.0	9	WHOQOL-BREF	Generic	A
Peymani M, 2006 [42]	2005	Persian	Tehran	Neuropathy	Cross sectional	Type 1, Type 2	304	>18	76.1	NA	Developed by research team	Specific	A
Peymani M, 2007 [43]	2004-2005	Persian	Tehran	Cardiovascular disease	Cross sectional	Type 1, Type 2	302	>18	76.1	NA	Developed by research team	Specific	A
Peymani M, 2008 [44]	2005	Persian	Tehran	Retinopathy	Cross sectional	Type 1, Type 2	178	>18	75.8	NA	Developed by research team	Specific	A
Rakhshandehru, 2006 [45]	2001-2002	Persian	Tehran	Health education	Quasi-experimental	Type 2 (100)	44	40-65	45.5	NA		Specific	U
Rasouli D, 2011 [46]	2008	Persian	Tehran	The predictors of HRQoL	Cross sectional	Patients with diabetic foot ulcer (100)	120	>45-65<	40.0	NA	Diabetic foot scale questionnaire	Specific	U
Safavi M, 2011 [47]	2009-2010	English	Ardebil	Health education	Randomised controlled trial	Type 2 (100)	123	30-70	50.8% (experiment), 51.6% (control)	NA	Farrell and Grant quality of life questionnaire	Generic	U
Sanjari M, 2011 [48]	-	English	Kerman	Foot ulcer	Case–control	Type 1 (11.4), Type 2 (88.6)	132	-	37.9	10.9	SF-36	Generic	A
Sayadi N, 2011 [49]	2007	Persian	Khuzestan	Open heart surgery	Case–control	Type 2 (38.8), non-diabetes (61.2)	80	35-75	65% (diabetic), 33% (non-diabetic)	4.3	SF-36	Generic	U
Shahrjerdi S, 2009 [50]	2008	Persian	Markazi	Physical excercise	Quasi-experimental	Type 2 (100)	27	>35	100	5.3	SF36, General Health Questionnaire	Generic	U
Shareh H, 2012 [51]	-	Persian	Fars	Perceived social support	Cross sectional	Type 2 (100)	50	NA	NA	NA	Multidimensional Scale of Perceived Social Support, WHOQOL-BREF	Generic	A
Taghdisi MH, 2012 [52]	2009	English	Golestan	Health education	Quasi-experimental	Type 2 (100)	78	NA	79.5	NA	WHOQOL	Generic	U
Timareh M, 2012 [53]	-	Persian	Kermanshah	Predictors of HRQoL	Cross sectional	Type 1 (4), Type 2 (96)	350	>18	58.3	NA	SF-36	Generic	U
Vares Z, 2010 [54]	2006	Persian	Isfahan	Predictors of HRQoL	Cross sectional	Type 1 (18.4), Type 2 (81.6)	310	>18	74	10.9	Iranian Diabetes Quality of Life questionnaire	Specific	A
Vazirinejad R, 2010 [55]	2007	Persian	Kerman	Diabetes	Historical cohort	Diabetes (45.1), non-diabetes (54.9)	224	<30->60	76.3	8.0	SF-36	Generic	U
Yekta Z, 2011 [56]	2009-2010	English	West Azerbaijan	Foot ulcer	Cross-sectional	Type 2 (100)	250	-	61.6	7.7	SF-36	Generic	A

### HRQoL instruments

Regarding the instrument used to measure HRQoL, 17 different instruments were used in the studies. 25 studies used a generic measure, 13 studies used a disease-specific instrument and 7 studies applied both generic and disease-specific instruments among the study population. 14 studies used the Short Form Health Survey (SF-36) and 10 studies employed WHO quality of life instruments (WHOQOL) to evaluate HRQoL in patients with diabetes. In 10 studies an Iranian questionnaire, developed by authors or research teams of those studies or adapted from other Iranian researchers, were applied.

### HRQoL in diabetes patients vs. non-diabetes

Six studies, which examined the effect of diabetes on HRQoL, compared HRQoL in people with and without diabetes and reported negative effects of both type 1 and type 2 diabetes on HRQoL.

### The effects of interventions

A total of 12 studies examined the effects of an intervention on HRQoL in people with diabetes. All studies, but one, evaluated the effects of educational interventions on HRQoL in people with diabetes and demonstrated improvements in HRQoL caused by these interventions.

### Diabetes-related complications

Six studies mainly examined the effects of diabetes-related complications on HRQoL in patients with diabetes and reported negative effects of these complications on HRQoL. In addition, among remaining studies, 9 studies included these complications as a predictor of HRQoL and found that these complications were associated with lower HRQoL.

### Other predictors of HRQoL

Association between HRQoL and some demographic, socioeconomic and clinical predictors were examined in the most studies. Except one study, all the other studies found a negative association between age and HRQoL. Moreover, in all studies except one, females had lower HRQoL than males. Better socioeconomic status (including income, education, employment) of individuals and/or their family was associated with better HRQoL. Better HRQoL was reported for married compared to non-married (single, widow) people. People with higher HbA1c generally had lower HRQoL. There were negative associations between blood pressure, blood lipid and HRQoL. Lower level of HRQoL was found among people with higher BMI. While most studies found a negative association between HRQoL and duration of diabetes, two studies reported a positive association. Smokers had worse HRQoL than their non-smoker counterparts. Two studies examined rural/urban disparities in HRQoL, but their results were not consistent, showing opposite results. In general, patients who were under diet treatment had better HRQoL than patients on drug or/and insulin therapies.

## Discussion

To our knowledge, for the first time, the current study has reviewed the results of 46 identified studies examining HRQoL among the Iranian patients with diabetes. The findings of this review showed that generally people with diabetes have worse HRQoL than their healthy counterparts. In addition, the findings indicated that diabetes-related complications have a significant negative impact on HRQoL among the diabetic patients in Iran. In general, associations between covariates and HRQoL in the reviewed Iranian studies were in line with their international counterparts.

The reviewed studies suffer from major methodological and reporting flaws which affected quality of their findings and limit their validity and generalizability. The reviewed studies mainly applied a nonrandom sampling method leading to possible selection bias. Moreover, calculation of sample size was unclear in the majority of the studies. Furthermore, while most studies were observational, univariate analysis was the main statistical approach used for data analysis and minimum effort was done to control for any imbalance in the covariates leading to potential confounder and selection biases. Among studies which employed multivariate analysis, some of the main confounding factors (such as diabetes-related complications and duration of diabetes) were not controlled for, raising possibility of confounder bias. In addition, these studies didn’t explain their limitations adequately and did not comment on the potential biases in their reported results. Although, generic instruments were used by the most studies, limitations of these instruments in capturing HRQoL in patients with diabetes were not fairly explained. Moreover, several studies failed to validate the instruments before putting to use in a new population and only referred to application of the instruments in a diabetic population in other countries or a general population in Iran. It seems that similar to few other settings [57], Iranian researchers have used the instruments applied in other studies without worrying about their content.

The results of the current review should be interpreted in light of few limitations. Firstly, although Persian databases used in this review consisted majority of the articles published nationally, there is a possibility that some studies may not be included in these databases. Secondly, as a wide range of instruments were used in the reviewed studies and the transparency of reported results was limited, it was not possible to apply statistical methods such as meta-analysis to test association between the covariates and HRQoL. Increasing the number of studies applying the same instrument and improving transparency of reporting results may make it possible to conduct a meta-analysis in future.

The previous systematic reviews mainly have focused on evaluating and comparing measurement properties of instruments used in examining HRQoL among diabetes patients [57–61]. In a review of HRQoL studies among people with diabetes in Nordic countries, Wandell [62] found that diabetes had a negative effect on HRQoL and being at older age, having diabetes-related complications, having lower socioeconomic status, being female and having weaker control of clinical risk factors were associated with lower HRQoL. These findings are comparable to the findings of the current review.

In conclusion, growing interests in evaluating HRQoL among people with diabetes were observed in Iran over the last decade. The findings of this review showed that people with diabetes had a lower HRQoL than healthy people. The findings also indicated that better socioeconomic status and better control of cardiovascular risk factors were associated with better HRQoL among the patients with diabetes. The reviewed studies suffer from major methodological and reporting flaws which limit the validity and generalizability of their findings.

## Authors’ original submitted files for images

Below are the links to the authors’ original submitted files for images. Authors’ original file for figure 1 Authors’ original file for figure 2
