# Supplementary figures and images for: Quality of life in people with diabetes: a systematic review of studies in Iran
Source: J Diabetes Metab Disord. 2013 Dec 19;12:54. doi: 10.1186/2251-6581-12-54 (PMC7968439; doi:10.1186/2251-6581-12-54)

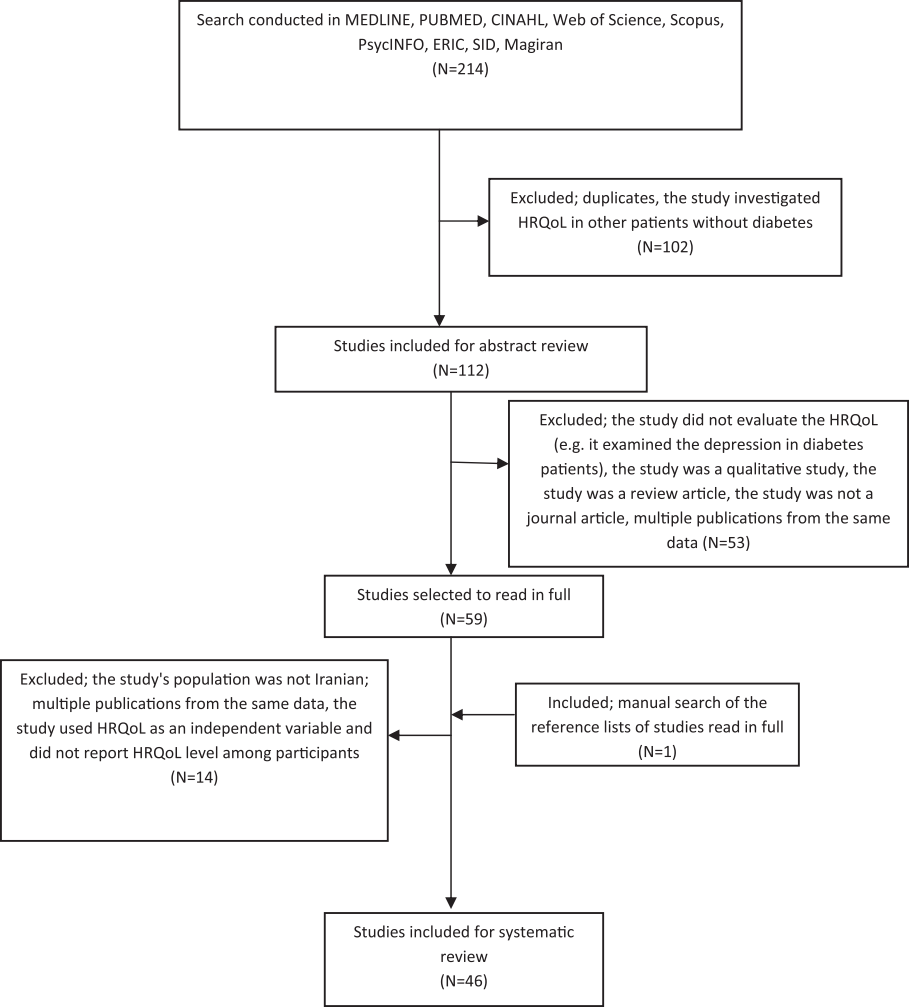

Supplement: Supplementary file 1 — Authors’ original file for figure 1 [file 40200_2013_186_MOESM1_ESM.pdf]

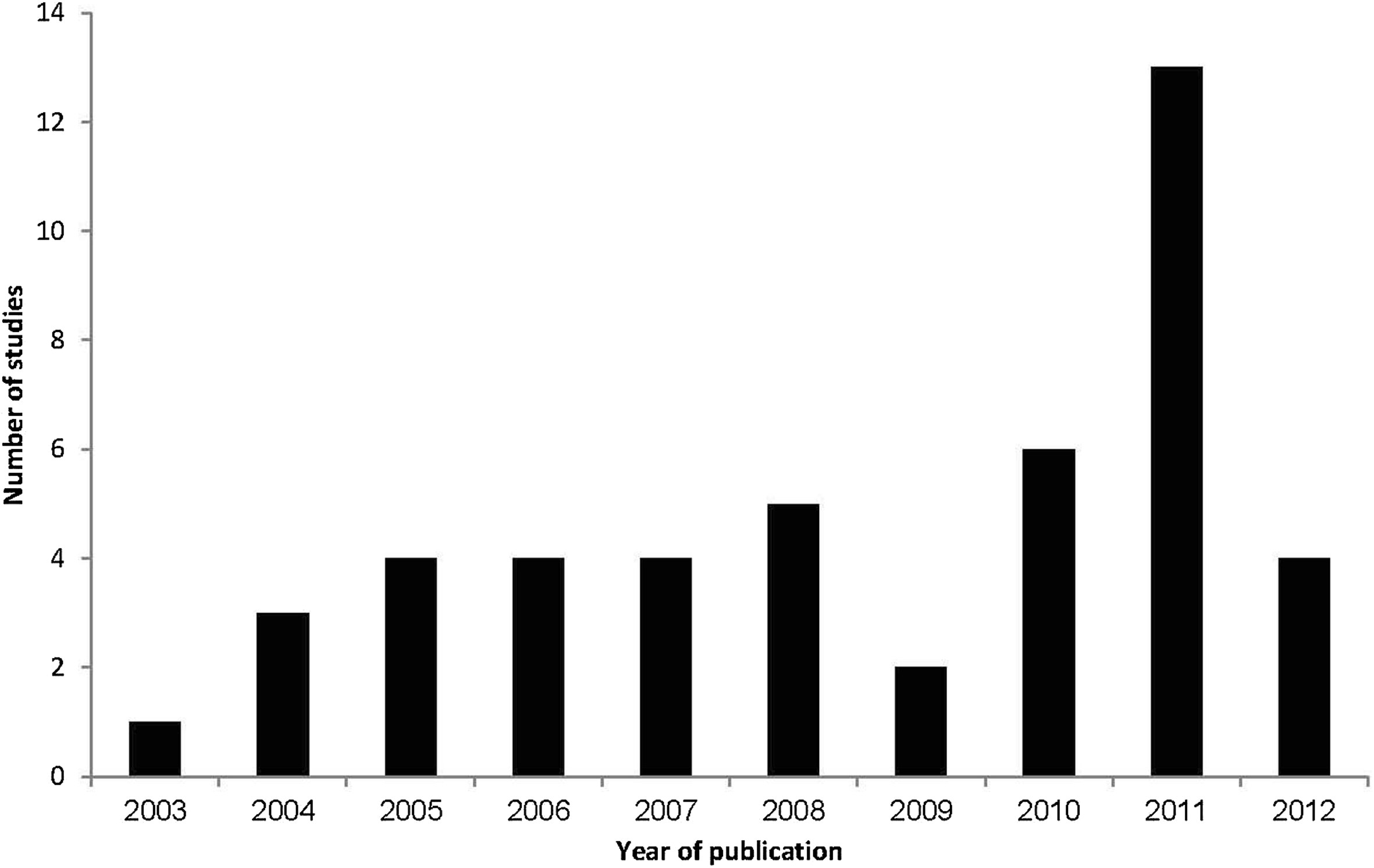

Supplement: Supplementary file 2 — Authors’ original file for figure 2 [file 40200_2013_186_MOESM2_ESM.tif]
